# Creating change in government to address the social determinants of health: how can efforts be improved?

**DOI:** 10.1186/1471-2458-14-1087

**Published:** 2014-10-20

**Authors:** Gemma Carey, Brad Crammond, Robyn Keast

**Affiliations:** National Centre for Epidemiology and Population Health, Australian National University, Canberra, Australia; Department of Epidemiology and Preventive Medicine, Monash University, Melbourne, Australia; Business School, Queensland University of Technology, Queensland, Australia

## Abstract

**Background:**

The evidence base for the impact of social determinants of health has been strengthened considerably in the last decade. Increasingly, the public health field is using this as a foundation for arguments and actions to change government policies. The Health in All Policies (HiAP) approach, alongside recommendations from the 2010 Marmot Review into health inequalities in the UK (which we refer to as the ‘Fairness Agenda’), go beyond advocating for the redesign of individual policies, to shaping the government structures and processes that facilitate the implementation of these policies. In doing so, public health is drawing on recent trends in public policy towards ‘joined up government’, where greater integration is sought between government departments, agencies and actors outside of government.

**Methods:**

In this paper we provide a meta-synthesis of the empirical public policy research into joined up government, drawing out characteristics associated with successful joined up initiatives.

We use this thematic synthesis as a basis for comparing and contrasting emerging public health interventions concerned with joined-up action across government.

**Results:**

We find that HiAP and the Fairness Agenda exhibit some of the characteristics associated with successful joined up initiatives, however they also utilise ‘change instruments’ that have been found to be ineffective. Moreover, we find that – like many joined up initiatives – there is room for improvement in the alignment between the goals of the interventions and their design.

**Conclusion:**

Drawing on public policy studies, we recommend a number of strategies to increase the efficacy of current interventions. More broadly, we argue that up-stream interventions need to be ‘fit-for-purpose’, and cannot be easily replicated from one context to the next.

## Background

Over the last forty years a new paradigm has emerged in public health demonstrating that social factors such as housing, employment, education and the urban environment are the strongest influences on population health [1, 2]. One of the key challenges for public health is how to effect change in these social factors when many of the most important influencers – such as government institutions – do not always appreciate the health consequences of their work, and continue to adopt a siloed approach to problem identification and solution [3, 4]. Creating a whole of government response, in order to break down these siloes, is seen as an imperative for addressing the social determinants of health.

The necessity of including the whole of government in the effort to improve the social determinants of health has long been recognised. Canada’s 1974 *Lalonde* Report, credited with kick-starting the health promotion movement, recommends that all government sectors become responsible for health promotion [5]. Similarly, the 1980 *Black Report*, a landmark review of health inequalities in the United Kingdom, recommended the Cabinet Office machinery be made responsible for reducing health inequalities. The *Black Report* argued that a reduction in health inequalities will be achieved “only if each department makes its appropriate contribution and this in turn, we believe, requires a better degree of co-ordination than presently exists” [6] (p 205).

Today, the model of government coordination sought by public health advocates has become more sophisticated but the central purpose remains the same: to have all relevant government departments included in all aspects of policy making and implementation on health inequalities. Thus, regularly, those that advocate - in policy, research or practice - for the adoption of a social determinants of health gaze are arguing for changes in the way that government processes occur as much as they are arguing for specific changes to government policies. For example, Marmot and colleagues have recently argued for greater integration across government: “Reducing health inequalities is clearly a task for the whole of government, locally and nationally. However, too often action has been limited by organisational boundaries and siloes” [7] (p 86).

The types of interventions – or changes – in government process sought by public health advocates can be termed ‘instrumental process-based interventions’ (IPIs). We include the word ‘instrumental’ because these interventions are not proposed as being inherently able to improve health but rather that their implementation will be instrumental in the creation of healthier policy. They are also process-based, not simply in their focus upon government processes, but also because the interventions are usually constructed as introducing new decision-making processes and do not have explicit target policy outcomes. Finally, we refer to these processes as interventions following Hawe and Potvin’s definition of an intervention as to ‘come between’ [8]. Some approaches to JUG, such as the Fairness Agenda and HiAP are each designed to ‘disturb the natural order’ of policy making by coming between traditional methods and typical outcomes (the NHS’s ‘Change Day’ provides an excellent illustrative example [8, 9]). IPIs, as we refer to them in this paper, are interventions based on networked approaches to governance and public management, which recognise interdependencies between different actors [10].

There are two IPIs appearing in the public health literature that have gained significant attention over the last three years and which have the potential to impact the social determinants of health at the government level: ‘fairness at the heart of all policies’ (or the Fairness Agenda, as we refer to it here); and increasingly well-recognised ‘Health in All Policies’ (HIAP). These IPIs draw on broader discourses of ‘joined up government’ (JUG), which are increasingly prevalent in the public policy literature.

The aim of the paper is to identify lessons from the existing body of evidence on JUG, which can help strengthen HiAP approaches currently being implemented. Joined-up government approaches to health exhibit considerable potential and any lessons which can be learned from previous experiments can improve effectiveness and avoid costly failure. To this end we conducted a meta-analysis of the research on JUG initiatives in the public policy studies literature. We argue that there is a need to carefully align the instruments used in IPIs with their goals and the contexts in which they are implemented. Drawing on the public policy literature, we recommend a number of strategies to increase the efficacy of current interventions.

### Joined up government

‘Joined up government’ or ‘whole-of-government’ approaches have emerged in many industrialised countries in the last twenty years as an attempt to grapple with ‘wicked’ public and social policy issues which implicate multiple government departments [11–13]. The term ‘joined up government’ was coined by the Blair Government (UK) with its focus on a ‘Third Way’ and a desire to address issues of social exclusion. Indeed the intractable, interconnected and ‘wicked’ problem of social exclusion remains a key focus of JUG [14, 15]. Other complex issues targeted by JUG include domestic violence [16], drugs and crime [17], homelessness [18] and poverty [19].

‘Joined up government’ is an umbrella term; how JUG is ‘done’ depends on the particular characteristics of the government of the day, its political imperatives and the nature of the problem(s) being addressed [20].

The management of complex networks, such as those targeted by JUG, is not a simple matter of diagnosing the problem and selecting the appropriate instruments (as one might find in discussions of hierarchical governance and management arrangements) [21]. As De Bruijn and Ten Heuvelhof emphasise, effective network management is a negotiated process [22]. Just as there is no authoritative actor who can implement management tools [23], there is no one actor with a sense of the problem(s) as a whole. The diagnosis of the problem will differ depending on vantage point within the network, while the solutions must work in and through the network and be tailored to the actors and their contexts to be effective [24]. Hence, the actors and goals of networks – and the nature of their interactions – are fundamental aspects of complex network management and governance.

A focus on networks means that JUG efforts must involve the “use of institutions and structures of authority and collaboration to allocate resources and coordinate and control joint action” [21] (p231). Through exploring instruments for multi-organisational, or network, management De Bruijn and ten Heuvelhof identify three levels at which change in complex networks are sought [22]:

- Instrumental - focuses on how governments seek to exercise legitimate authority by altering dependency relationships. Governments use tools that increase or restructure networks.
- Institutional – focuses on establishing the rules of engagement as well as organisational frameworks that can set the stage for ongoing interactions and strategy development
- Interpersonal – the aim is to shape the interactions between a range of actors to generate innovative responses [23, 25, 26]. In this bottom-up approach, the focus is not on attaining or delivering pre-set external goals but about contributing to and providing conditions for the process of finding a common purpose among a diverse set of interests.

These ideal types help to conceptualise the level at which change is pursued, however, in practice change may be pursued on a number of levels, using a range of instruments and/or processes.

A number of attempts have been made to identify principles of best-practice for JUG [27–29]. Common features are new organizational units and intergovernmental councils that aim to create integration across ‘siloed’ departments, along with the pursuit of broad-based goals articulated by communicative instruments [30]. Through these mechanisms, whole of government approaches are seen as promoting agreement, collaboration and the straightening out of inconsistent policies [14, 31].

Richards notes a ‘glibness’ in the way JUG is used, which disguises the major structural and systemic changes required within government [14]. Amongst public administration scholars there is growing interest in understanding how whole of government approaches are created and whether they are appropriate or effective in combating the problems they set out to address [31–33]. Increasingly, there is a focus on developing ‘fit-for-purpose’ strategies [34, 35]. Many failed JUG experiments have been the result of incompatibility between the goal, the instruments used to pursue integration and the context in which change is sought [34].

In short, the term ‘joined up government’ masks the diversity of approaches and the complexities of the challenge and there is limited empirical evidence base concerning the implementation and effectiveness of JUG. A growing body of research demonstrates the need for compatibility between goals, the instruments and processes used to achieve them and their level of deployment (i.e. within government or between government and other entities) and the context in which change is pursued – emphasising ‘fit-for-purpose’ strategies [34]. Overall, a joined up approach is no guarantee of success – it is difficult to achieve and highly variable in its outcomes [20, 34].

Public health interest in JUG can be traced back to the eighties and has intensified in the last decade as a way to address the social determinants of health [36, 37]. JUG has also been advocated for more broadly in recent reports such as the Strategic Review into Health Inequalities in the UK [7]. In their Task Group paper for the review, Whitehead and colleagues draw strongly on the public policy literature, suggesting that systems for addressing inequalities in health need to take action at all levels of government, along with civil society and the corporate sector [38]. They also cite changes to accountability through local and national targets, shifts in culture and organisational restructuring as important features of joined up approaches to address the social determinants of health.

These arguments are based on the understanding that social determinants of health include institutions and services from outside the health sector and as such government departments other than the health department have a considerable influence on population health. However, these departments are understood to operate separately from one another, in departmental ‘silos’. The central aim of JUG approaches is to break down these siloes so that decision-making in, for example, the Transport Department occurs in concert from representatives of the Health Department. In this way it is hoped that new transport policies will be introduced which are health promoting rather than health damaging.

In addition to non-health departments having an effect on health, the push for JUG has been based largely on the understanding that ‘wicked’ social and health problems are interconnected in nature. That is, social and health inequalities are understood to emerge from interconnected problems and therefore the logical response is the creation of interconnected decision-making through JUG [7].

More recently two prominent interventions (based on principles of JUG) have emerged in the social determinants of health literature, that proponents – including the Strategic Review of Health Inequalities and the World Health Organisation – are vehemently advocating for. These new interventions are the primary focus of this paper and are discussed in more detail in the following section. Governments have generally sought out joined-up solutions to their own policy problems. When it comes to the social determinants of health, existing IPIs have been designed by those outside of government, who are now advocating for their take up. For this reason, we have termed these IPIs ‘interventions’.

### IPIs for the social determinants of health

In this section we provide an overview of the two IPIs examined in this paper. We discuss and critique the goals and instruments used in each to create change. Table 1 provides an overview of instruments and their objectives.

**Table 1:** **IPIs, instruments and objectives**

IPI	Instruments & tools	Objective
**Fairness Agenda**	Inter-departmental committees/taskforces	*Integrated cross- government policy*
	Health Equity Impact Assessments	*Adoption of policies that reduce health inequities*
	Policy Current	*Cultural & institutional change*
**HiAP**	Taskforce backed by political leadership	*Make health the central goal of policymaking*
	Health Lens Analysis	*Adoption of policies that improve health*

#### Fairness Agenda

The Strategic Review of Health Inequalities in the UK (the Marmot Review) is one among a series of recent reviews chaired by Sir Michael Marmot regarding the political action required to narrow health inequalities. The wellknown *Closing the Gap in a Generation* by the WHO Commission on Social Determinants of Health, and the less widely reported *Report on Social Determinants of Health and the Health Divide in the WHO European Region* by the WHO Regional Office for Europe offer analyses at the global and regional level. The Marmot Review (perhaps better termed the UK Marmot Review), being a domestic review, is most detailed in its outline of specific interventions for the reduction of health inequalities and fits into a history of UK reports on health inequalities. Like the Black and Acheson Reports which preceded it, the Marmot Review calls for a whole of government response to health inequalities. The whole of government intervention is to have two key aspects. First is ‘robust political leadership’ from ‘the Secretary of State for Health with an explicit cross-government remit to deliver on health inequalities’. This leadership is to be ‘supported by the appointment of joint, multi- skilled teams working across all relevant government departments to facilitate integrated cross-government policy under the direction of a single lead director with overall authority and responsibility’. The second aspect of the intervention is the introduction of a ‘health equity impact assessment’ which all government policies and strategies are to be subject to. The Fairness Agenda therefore seeks structural integration (between departments) and process integration (through the use of health impact assessments), using a centralised model (i.e. the agenda, tasks and processes are set by a taskforce).

Supplementing the proposed new government processes is a policy narrative of ‘fairness’, which is to be at the heart of all policy and delivery [39–41]. Drawing on the political philosophy of Amartya Sen ‘fairness’ is here seen as a way to create change in diverse policies and whole of government responses by encouraging policymakers to concentrate on enhancing the capability of the population to be healthy [42, 43].

Like ‘social inclusion’ and ‘social exclusion’, concepts of fairness are thought to provide a goal for all departments and areas of government. Here, fairness is being advocated for use as a ‘policy current’ or ‘policy narrative’ [44]. Emerging from a pragmatic and business-like government view (in some countries known as the Third Way, in others as the Purple Coalition - Red for social-democrat and Blue for Liberal) [45], policy currents are said to sit ‘above’ policies and act as a rallying call, directional pointer and broad benchmark for change [46]. The limited available literature on policy currents describes them as fluid, guiding policies or principles that move across departmental boundaries [46]. Policy currents are equivalent to emerging concepts discussed in the organisational management literature and now being used in health care settings [9]. For organisational change, Bartel and Garud encourage the use of ‘innovation narratives’ [47]. Like policy currents, innovation narratives promote the coordination of people and ideas across organisations, shaping practice through the power of language and the story about an innovation; such as a new approach to policy. Policy currents are an attempt to create essential ‘cultural-institutional’ change – uniting stakeholders behind a common goal [12]. These communicative instruments are not intended to modify behaviour as such, but to bring about change in how actors perceive the problem and shift people’s values [10].

#### Health in All Policies

The ‘Health in All Policies’ (HiAP) is public health intervention that attempts to create JUG with an explicit health focus [48]. HiAP has emerged from the health promotion literature of the nineteen-seventies and eighties [49], typified by the Alma Ata Declaration [50] and the Ottawa Charter for Health Promotion [51]. These documents, and the sizeable literature they inspired, seek to create supportive environments in which healthy choices are possible and easier for citizens so that people are enabled to lead healthy lives [52] (p5). This literature, collectively referred to as dealing with ‘healthy public policy’, encompasses a wide variety of strategies and interventions.

**Figure 1:**
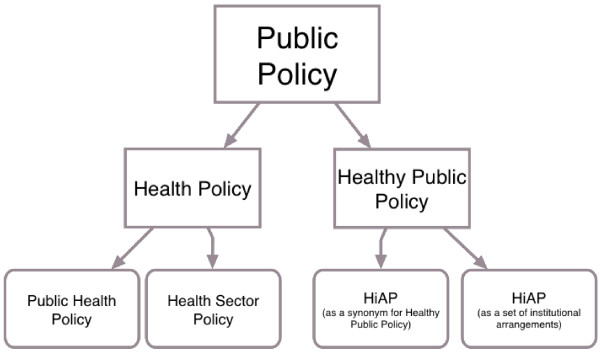
**HIAP and healthy public policy.**

While HiAP has been used interchangeably with healthy public policy the term is also used to describe one specific strategy to enhance population health by introducing health considerations into the decision-making of non-health sectors [52]. This latter version of HiAP, as a set of institutional arrangements for delivering JUG, is the form addressed in this paper. Figure 1 displays where HiAP is situated in the health policy literature.

There are some differences of emphasis even among the institutional versions of HiAP. The initial formulation in Finland, taking its inspiration from the successful North Karelia programmes [53], emphasises initiatives at the community level which form a package of measures that simultaneously promote health, welfare and productivity [52, 54]. In contrast, the form of HiAP pursued in South Australia, (which has received much attention internationally [55]) is embedded in the government bureaucracy making it a more centralised, top-down approach to joined-up government. Each approach seeks the integration of health and other government sectors, but where the Finnish model is fairly vague regarding how this will occur, the South Australian model is very specific: its chief tool is a ‘Health Lens Analysis’, supported by a HiAP Unit in government and applied to all new government policy [56–58].

The Health Lens Analysis operates similarly to the more widely discussed Health Impact Assessment (though there are some practical differences, see [59] for further discussion) and provides the primary means of incorporating health into other government sectors. New policy proposals are to engage the HiAP Unit at an early stage of development so that health considerations are present throughout policy development. It is hoped that this process will improve the ‘healthiness’ of eventual policies. Available documentation describes a process in which health and health equity are incorporated into policies in a collaborative and iterative fashion. There is, however, no guidance regarding potential conflicts between departments and the HiAP Unit or how to resolve them. If a proposed policy ‘fails’ the Health Lens Analysis the HiAP Unit is not backed by any legislative power and does not appear to have the authority to prevent a policy from being adopted and implemented. Hence, this approach to HiAP relies strongly on relationship building [49].

In South Australia HiAP was initially supported by a HiAP Unit situated in the Department of Health and an Executive Sub-Committee of Cabinet which provided a ‘mandate for the application of HiAP to…the work of non-health agencies’ [49] (p 194). At the time of writing, with the disbanding of the Sub-Committee in 2012, the HiAP health lens is spread across seven newly created sub-committees matching seven new Cabinet Strategic Priorities. Despite the institutional changes, HiAP has continued to function. The centralised governance structures are said to be a cornerstone of the approach [49]. In South Australia, HiAP has been implemented in areas such as water sustainability, digital technology, migration and transport [49, 58]. At present, there is little peer-reviewed empirical work concerning HiAP and its effectiveness. One exception is the study published by Lawless et al. who provide qualitative insight into some aspects of the process of adoption in the South Australian context so far [60].

## Methods

The aim of the study is to identify lessons from the exiting body of evidence JUG, which can help strengthen IPIs currently being implemented, through a meta-analysis of joined-up government initiatives. What can be learnt from the health policy literature has been discussed elsewhere and is not the focus of this paper [24, 49, 61].

Bacchi [62] has argued for the importance of research synthesis for policy: for policy, meta-analysis provides a forum by which disparate empirical studies can be reduced to a common metric. At present, there are no agreed upon methods of qualitative research synthesis, and debate in this area has continued for some time [60, 63]. Thematic approaches to meta-analysis seek to uncover concepts and their meanings from the data (rather than pre-determining them), using interpretive approaches to ground the analysis of that data (i.e. existing studies).

Thematic approaches are useful for hypothesis generation and explanation of particular phenomena, though provide less of a picture of the context and quality of the individual studies [63]. As McDermot suggests, the epistemological and methodological debates and tensions concerning qualitative meta-analysis center on the contextual nature of qualitative research: “What this means is that we cannot assume that concepts, experiences and practices have homogenous meanings, which stay constant across time and place; different contexts support a variety of meanings” [60] (p11). This approach, however, is to deny the generalizability of qualitative research [64]. While a qualitative case study is a comprehensive examination of a single example, it can provide ‘trustworthy’ information about the broader class to which it belongs [65]. To claim that generalisation is not possible is to deny the transferability of any shared meanings or generative mechanisms [60]. Hence, the meta-analysis provided here presumes that it is both possible and desirable to seek out, and synthesise, the lessons that emerge from individual qualitative case studies.

**Figure 2:**
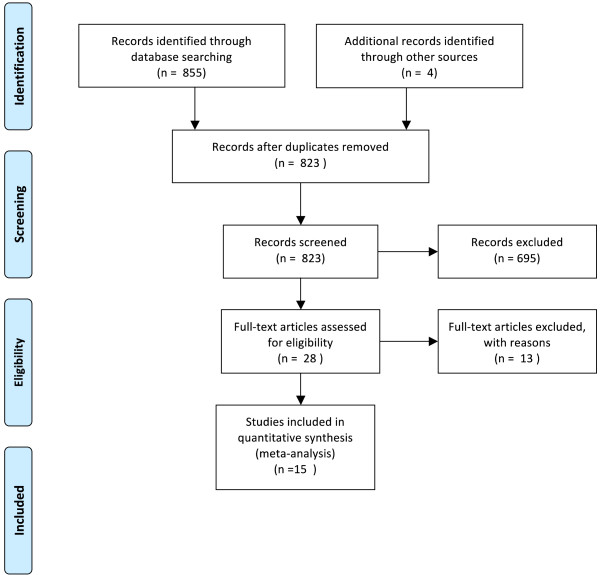
**PRISMA flow chart.**

In order to identify relevant empirical research on JUG, searches were conducted in Expanded Academic, Academic Complete, JSTOR, Web of Science and Science Direct between 1990 and 2014. Prominent journals in the field, including the International Journal of Public Administration and International Public Management Journal, were also searched independently. Search terms included health in all policies^a^, joined up government, joined up governance and whole-of-government. Results of the review are shown in the PRISMA flow diagram in Figure 2 [66].

In total, 823 papers were identified once duplicates were removed. Abstracts of these studies were screened for studies that were empirical evaluations of a past or existing national-level JUG initiative. To be classed as an empirical evaluation studies must have collected qualitative or quantitative data on the success of an initiative according to any indicator. Eleven further studies were excluded for either not collecting empirical data (described in the abstract as a ‘case study’) or for addressing international governance.

Sixteen empirical studies were identified for inclusion. The remaining 16 studies were subject to further analysis of their quality, using a framework adapted from McDermott [60], upon which one study was excluded due to poor quality. The remaining 15 studies comprised of case study analysis, or comparative case study analysis, of joined-up government initiatives. A case study is an in-depth study of a single unit, or a group of units, where the researcher’s aim is to elucidate features of a larger class of similar phenomena [67]. Case studies are an important part of social inquiry, though often ranked low on the ‘hierarchy of evidence’. Case studies have provided critical insights in areas such as clinical research, psychology, public health intervention research and through use as part of natural experiment study designs [65]. For researching public policy, case studies are an important method given the constraints of using other research designs (such as randomized control trials) in this context [36, 37]; in public policy, knowledge of policy design, implementation and outcomes are all built from case study research, using the ‘natural generalization’ of case studies [65].

From these studies, characteristics associated with success or failure of JUG were identified. Guided by the work of Keast [34], these characteristics are organised into five categories, reproduced in Table 2.

**Table 2:** **Five factors aiding joined-up approaches and how these compare to HiAP and the Fairness Agenda**

	Factors found to aid joined up approaches	HiAP	Fairness Agenda
**Operational Level**	Target multiple levels: [33, 34, 68–76]	Government – managerial	Strategic government
	Strategic government		
	Managerial		
	Practitioner		
	Community		
**Top-down/ Bottom-up**	Top-down & bottom-up [33, 34, 68–76]	Top-down	Top-down
**Nature of control**	Decentralised [33, 34, 68, 70, 71, 76–78]	Centralised	Centralised
**Focus**	Designed based on both the purpose and the context [20, 34, 70, 71, 74]	Embedding health in all policies	Promoting equity
**Instruments & their functions**	Fulfil a range of functions depending on objectives. For example:		
	Governance & structure (e.g. committees/ taskforces, creation of shared leadership) [34, 68, 69, 71, 72, 74–76, 78]	Governance & structure: *interdepartmental committees/taskforce*, *leadership (Taskforce located in Premier & Cabinet)*	Governance & structure: *interdepartmental teams*
	Managerial changes (e.g. to improve relationships) [73, 74, 77–80]	Managerial: *efforts to improve relationships between departments*	Adjusted processes: *Health Equity Impact Assessments*
	Adjusted systems, processes & finances [34, 69–72, 77, 81]	Adjusted processes: *Health Lens Analysis*	Culture: *policy current, political leadership*
	Cultural & institutional change [20, 34, 70, 71, 75–78, 80]		
**Membership**	Reflects the multiple levels targeted for change [20, 33, 34, 68, 70–75, 77]	Government departments	Government departments, politicians

## Results and discussion

A small but important body of empirical research into JUG exists. This research highlights critical factors that must be addressed to maximise the likelihood that JUG will be successful.

Table 1 provides an overview of features found to promote JUG identified in the public policy literature, against which we have compared HiAP and the Fairness Agenda. Five areas of concern are identified from the public policy literature which provide an indication of success: operational level, nature of control, top/bottom focus, instruments and membership. In the following discussion we examine each of these factors and the insights that can be gained from the public policy literature.

Importantly, both IPIs seek high-level political support, which as been found to be a crucial ingredient for the success of JUG [30]. Less favourably, however, they, take a top-down and centralised approach, predominately concerned with change within government. While these IPIs have been intentionally designed to focus on creating change in government, JUG initiatives have been found to be most effective when they work at a multiple of levels both within and external to government [34]. Successful initiatives also tended to engage non-government actors at the local level (such as non-government organisations) in collaborative (not just contractual) working arrangements, based on a high degree of mutual trust. This is because governments are reliant on individuals, groups and organisation that exist within the policy environment, but are external to government. Research into joined up approaches within the public policy literature indicate that initiatives need to engage this broad, and dynamic, set of actors. As Keast argues, “while top-down approaches are important to set priorities and push through a joined-up ethos, cooperative relations on the ground may prove to be more important in the long run” [34] (p229).

Centralised approaches, have also been found to have limited effectiveness in promoting change within government itself, unable to breakdown programmatic and departmental silos [20, 34, 35]. In particular, strong leaders were found to be as critical at all levels. It appears that without champions at each level, joined-up ethos ‘washes-out’ and fails to take hold. While political mandates and visionary strategy needs to be secured at the top, in terms of integrated, collaborative practice those at the ‘bottom’ or street level are often more advanced, as joined-up working is often demanded in case management and responsiveness to local issues [68, 69]. Hence, initiatives need to be both ‘top-down’ and ‘bottom-up’, which is more consistent with the Finnish approach to HiAP.

As indicated in the ‘Focus’ row in Table 2, successful joined up initiatives are designed on both the purpose (what they hope to achieve) and the context (the system in which change has to occur, including structures, values and norms). The literature on JUG emphasises that IPIs need to be ‘fit-for-purpose’. This suggests that the implementation of these IPIs within different levels of government (i.e. local, state and national) as well as across different countries is likely to be problematic. The compatibility of these interventions with different structures of government found at different levels and in different countries (including the differing influences actors outside of government have on policy) is unknown. This is particularly problematic in the case of HiAP because it is a highly structured intervention initially designed within a relatively small government. The transferability of HiAP from places such as the South Australian State Government to more complicated bureaucracies found at the federal level (and also at the state level internationally) is likely to be limited and require significant amounts of context-adaptation. While adaptation to local contexts is noted in the HiAP literature, the fundamental goal of placing health at the forefront of all policy areas through the use of health impact assessments remains the same.

The emphasis placed on context and the adaptability of interventions found in the public policy literature is consistent with public health research into community interventions. In community intervention research, communities are increasingly seen as ‘complex systems’ of processes and events [82]. Through this lens, interventions are conceived as “sociocultural events”, which interact with local contexts [83]. These contexts are understood to be made up of norms, values and existing practices and beliefs [84]. This ‘context’ has been found in some instances to disrupt, and in others amplify, change – indicating that interventions must be aligned to their contexts in order to be successful [85–87].

With regard to instruments, both IPIs use interdepartmental committees to create integration and consensus around the importance of health (in the case of HiAP) and equity (in the case of the Fairness Agenda). The use of interdepartmental working groups is a common feature of JUG [33, 34]. These ‘taskforces’ are intended to breakdown organisational silos, remove contradictions and dysfunctions in existing structures, and promote holistic and innovative thinking [13]. However, these types of committees and taskforces have been found to *limit*, rather than facilitate, collaboration [10, 20, 34, 88]. Interdepartmental groups charged with leading JUG that have no formal authority in other departments generate limited change at best, and at worst, can create “serious dysfunction” [70]. Pollit and James warn against creating new sets of organisational enclaves in the pursuit of integration [31, 88]. Developing interdepartmental committees can end up creating new teams and administrative structures that are not well integrated with existing departments. Here, departments continue to carry the burden of accountability and implementation, while interdepartmental teams generate ideas, but lack the implementation capacity or accountability mechanisms to get things done. This makes them vulnerable to budget cuts in the face of cost pressures, as they are perceived to be ineffective and a drain on departmental resources [70]. This is important because joined up action carries high costs, including money, time and energy, along with ‘policy costs’ resulting from compromises that must be accepted for collaborative efforts to work [9]. This makes them easy targets. To avoid this, interdepartmental groups need to be supported by strong structural links to the departments they are working with through, for example, accountability mechanisms.

With regard to ‘softer’ tools such as policy currents, the evidence is sparse. However, research into JUG has highlighted the critical role of creating cultural and institutional change [12], which policy narratives such as ‘fairness’ can help to achieve. Exworthy and Hunter have recently questioned the ability of ‘health’ to gain traction as a central goal of government [89] (see also [90]). Policy outcomes emerge from interaction amongst different stakeholders and decision makers, all of whom are pursuing solutions to their own problems, rather than one ostensible problem like health [30, 91]. Moreover, as Lindblom contends, policy is often made through the interaction of a plurality of partisan individuals, meaning it is a highly negotiated process in which ‘neutral’ evidence of problems, such as the social determinants of health, play a minor role [91]. Having said this, health has historically been seen as an acceptable area of government intervention and may offer policymakers in other areas new avenues for pursing change [92].

### Creating more effective interventions

Public policy research into JUG has found that the instruments used to create integration and collaboration are often inadequate or inappropriate. Two underlying factors have been identified that explain this shortcoming: [1] IPIs often lack a ‘supportive architecture’, and [2] there is often a fundamental mismatch between the goals they aim to achieve, the mechanisms used to achieve them and the level at which they are deployed.

Existing research indicates that interventions like HiAP and the Fairness Agenda – that seek integration across government coupled with changes to systems and processes – require a ‘supportive architecture’ within government [70]. O’Flynn and colleagues warn that “without careful attention to, and investment in, creating [supportive] architecture, most attempts at JUG are doomed to fail, as the power of embedded ways of doing things restrains innovation and undermines cooperation” [70] (p253). In particular, initiatives that seek to create systems change (like HiAP and the Fairness Agenda) require high degrees of collaboration and integration, which in turn need to be supported by stricter mechanisms and arrangements than those only seeking to share information.

In Table 3 we outline the different elements of a supportive architecture. While IPIs do not need to have all of these characteristics to achieve some success, each has been found to be associated with positive outcomes [30]. We have divided these elements into ‘hard’ and ‘soft’: hard elements pertain to structure, while soft elements are aimed at creating cultural and institutional change. Some aspects of this supportive architecture are emerging in the grey literature on HiAP, including a political mandate for change and dedicated resourcing [93].

**Table 3:** **Elements of a supportive architecture**

Hard	Soft
A mandate for change [33, 34, 72, 76]	Deliberate and strategic focus on collaboration [34, 68–76, 78, 80]
Decentralised control [33, 34, 68, 70–72, 74, 78]	Training and skill development [34, 68, 70, 71, 75, 76, 78]
Accountability and incentive mechanisms [34, 69, 70, 72, 75]	A call to action or a rallying point [34, 68]
Dedicated resources (including flexibility in the way they are used at different levels) [34, 71–74, 76, 78]	Information sharing [70, 71, 74, 78, 80]

While both IPIs have some elements of a supportive architecture in place (such as a mandate for change), they could be strengthened by the addition of accountability and incentive mechanisms, which would support integration and create formal relationships and structures between interdepartmental committees and departments, organisations or individuals targeted for change. These include formal mechanisms, such as performance-based accountabilities built around output and outcome measurements, through to more informal accountability arrangements, such as dialogue [94, 95]. This combination of formal and informal can help to build a system of responsibility, where informal mechanisms such as dialogue support traditional performance management arrangements, maintaining a ‘creative tension’ between achieving goals and outcomes, and ensuring flexibility and innovation [95]. The overall goal of accountability arrangements is to ensure a “web of multiple, overlapping accountability relationships” that are functional and effective [94] (p197).

## Conclusion

Drawing on the trend towards JUG, IPIs have significant potential to reconfigure the way governments ‘do business’, helping to facilitate integrated policy design, implementation and service delivery. However, examples of successful joined up approaches are uncommon; in many ways, both attempts at creating JUG and the research that supports it is in its infancy.

We found that existing IPIs for the social determinants of health would be strengthened by stronger accountability and incentive mechanisms to support integration. Interestingly, the public policy literature suggests that some JUG instruments, such as interdepartmental groups (which have become a mainstay in the field) may actually limit collaboration.

There are some notable limitations to this study. We have chosen to review public policy literature in order to present a new perspective on public health research and, as a result, public health literature on joined-up governance has not been included (see, for example, [96]). This includes recent research on health public policy and health impact assessments, which similarly are attempting to bring conceptual clarity to the interface between public health and public policy [97, 98]. Similarly, the limited – though significant – research on Health Action Zones has not been considered here [99]. Finally, the review is limited to peer-reviewed sources. This has necessarily excluded monographs and the vast grey literature on JUG. We have accepted this limitation because, without peer-review, it is not possible to verify the studies’ quality, though we acknowledge that more might be learnt from this literature.

To move the field forward, more collaborative research is needed with public policy researchers, particularly to identify what aspects of a ‘supportive architecture’ are likely to be effective in different contexts. More broadly, some conceptual questions remain, such as how a targeted intervention (reducing social disadvantage in education) can be achieved by the application of the instrumental intervention (including Health Department officials in the making of education policy decisions)? As MacCathaigh and Boyle lament, “we still have too little joined up thinking about JUG” [100] (p220).

## Endnote

^a^No empirical evaluations for Health in All Policies were found. An evaluation of the South Australian approach is underway but has not published results. For these reasons results for the ‘health in all policies’ searches have not been included in the diagram.

## Abbreviations

JUG: Joined up government
HiAP: Health in All Policies
IPI: Instrumental process intervention.
